# What lessons can be learned from studying the folding of homologous proteins?

**DOI:** 10.1016/j.ymeth.2010.06.003

**Published:** 2010-09

**Authors:** Adrian A. Nickson, Jane Clarke

**Affiliations:** University of Cambridge, Department of Chemistry, MRC Centre for Protein Engineering, Lensfield Road, Cambridge CB2 1EW, UK

**Keywords:** Protein folding, Φ-Value, Protein families, Homologous proteins

## Abstract

The studies of the folding of structurally related proteins have proved to be a very important tool for investigating protein folding. Here we review some of the insights that have been gained from such studies. Our highlighted studies show just how such an investigation should be designed and emphasise the importance of the synergy between experiment and theory. We also stress the importance of choosing the right system carefully, exploiting the excellent structural and sequence databases at our disposal.

## 1 Introduction

Studies of the folding of homologous proteins have been gaining in popularity since the first explicit studies over 10 years ago. The inspiration for such studies comes from structural and sequence family databases, such as SCOP [1] and Pfam [2]. Initially the questions asked were relatively straightforward: do all proteins with the same structure fold via the same pathway? Are residues important for folding highly conserved? How can differences in the kinetics of folding be explained? In Table 1 we list all folds where more than one protein has been subject to detailed analysis of the folding pathway. In some cases proteins are closely related, with significant sequence identity (such as the immunity proteins, ∼60% identical); in others, only structure is conserved (for instance the immunoglobulin-like domains where proteins from different superfamilies have been compared); in other cases, in a twist to the tail, circular permutation has allowed the importance of chain connectivity to be investigated in proteins with identical sequence (such as the studies on the ribosomal protein S6).

Perhaps the most important insights into the folding field from studies of homologous proteins, have come from investigating *differences* between related proteins – proteins from the same family which have different folding mechanisms, proteins with different kinetic properties and those with completely different folding pathways.

The first importance of these studies is that they have allowed insight into some of the fundamental questions about protein folding mechanisms. These experimental studies have been particularly powerful when they have gone hand-in-hand with computational and theoretical work on folding. In the first part of this work we show how studies of the folding of families of proteins have been vital in developing our understanding of the relative importance of topology, sequence, entropy/enthalpy balance and secondary structure propensity in determining folding mechanisms.

In the second part we show how theoretical studies are adding to this work and highlight some very recent studies that show the power of this family approach to study protein folding: insights which would not have been possible from studies of individual proteins in isolation. We show that comparative studies have allowed rational design of folding pathways and altered kinetics.

Finally we address the design of studies of the folding of protein families.

## 2 Folding pathways and mechanisms

Although the ‘protein folding problem’ could theoretically be solved using a brute-force approach, where homologues to every conceivable protein sequence and structure are studied, a more subtle approach is to discover the pathways by which proteins attain their native state. This method also avoids the potential trap that proteins with very similar primary sequences can fold to different three dimensional structures [3,4].

As Valerie Daggett cogently observed: “To map [a] folding reaction we need to characterise all states along the way – native, transition, intermediate and denatured – as well as the mechanism of conversion between them” [5]. This feat has been achieved for a small number of proteins, by combining experimental studies on the free-energy maxima and minima with all-atom molecular dynamics simulations to add the remaining detail [6–8]. In addition, recent advances in NMR have made it possible to gain residue specific information about the structure of a polypeptide chain as it collapses towards the native state [9,10].

However, most of the information about protein folding comes in the form of a ‘snapshot’: an experimentally determined structure of a specific state on the folding pathway. In particular, hydrogen exchange studies are often used to probe the structure of kinetic intermediates [11], whilst Φ-value analysis [12] is extensively used to elucidate the structure of the major folding transition state (see Table 1 and references therein).

**Fig. 1:**
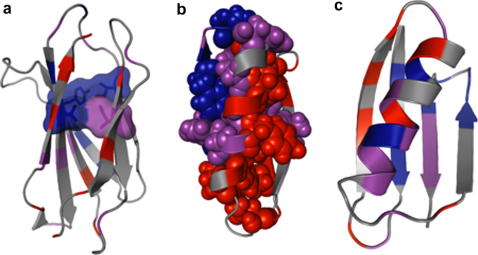
Example Φ-value patterns and how they illustrate aspects of the folding pathway. In each case the Φ-values are mapped onto a cartoon representation of the native state, with high values coloured blue, moderate values in magenta and low values in red. (a) TNfn3 [18]: the highest Φ-values are predominantly found in the central β-strands, and decrease away from a common-core ring of interactions, known as the ‘key-residues’, which set up the complex Greek-key topology of the native state. (b) Protein L [41]: the symmetry of the native state is completely broken during folding, with a high degree of structure in the C-terminal β-hairpin but virtually no structure formation in the N-terminal hairpin. (c) Protein G [42]: in contrast to Protein L, this transition state exhibits significant structure in the first β-hairpin, and little structure formation in the C-terminal hairpin.

Although these snapshots are not able to detail the exact order in which residue contacts are formed and consolidated (with a few exceptions [13]), an analysis of their structure is certainly able to identify contacts that must form early in the protein folding reaction and those which do not form until much later (see Fig. 1).

In order to facilitate a comparison between different folding pathways, the Φ-value pattern (or equivalent) is often used to determine a folding **mechanism** for the reaction. The terms ‘folding pathway’ and ‘folding mechanism’ are often used interchangeably in the literature, but here we are highlighting the distinction since a variation in one does not automatically imply a difference in the other:

• The pathway provides a complete description of the folding of a protein, stating the temporal order in which contacts are formed and consolidated as the polypeptide chain moves structurally towards the native state.
• The mechanism is a method of classification, and is a simplification of the pathway. It describes the relative abundance of secondary versus tertiary structure as the protein folds.

**Fig. 2:**
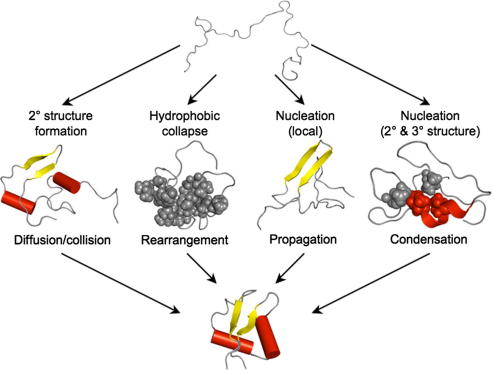
A description of the four ‘classical’ folding mechanisms. (1) The framework model [164] suggests that local elements of secondary structure form first. These then diffuse together, collide and adhere to produce the correct tertiary structure in the rate determining step. (2) The hydrophobic collapse model [165] implies that a protein collapses rapidly around its hydrophobic side-chains, and then rearranges from the restricted conformation of this ‘molten-globule’ intermediate. (3) The nucleation propagation model [166] states that local interactions form a small amount of native secondary structure, which acts as a nucleus for the outward propagation of further native structure. (4) The nucleation condensation model [167] suggests the presence of a metastable nucleus that is unable to trigger folding until a sufficient number of stabilising long-range interactions have built up. Once this occurs, the native structure condenses so rapidly that the nucleus is not yet fully formed in the transition state.

The folding mechanism is particularly useful when analysing proteins with markedly different native state topologies where no direct comparison of the folding pathway is possible. There are four ‘classic’ folding mechanisms, as described in Fig. 2.

The following sections use examples to illustrate some of the important results that have been achieved by studying the folding pathways and mechanisms of homologous proteins.

### 2.1 Proteins that fold with the same mechanism through similar pathways

The immunoglobulin-like (Ig-like) β-sandwich fold is one of the most populated [14] and all members exhibit a complex Greek-key topology, albeit with slight variations in the number and arrangement of β-strands [15]. Four of these domains, from two different superfamilies, have been subject to a detailed protein engineering study as part of the ‘fold approach’ [16–19]. In every case the proteins were found to fold through a nucleation–condensation mechanism where the obligatory folding nucleus comprises a set of buried hydrophobic residues in the B, C, E and F strands that form a ring of interactions in the core (see Fig. 1). These contacts are sufficient to establish the complex topology of the native state and, once formed, the rest of the polypeptide chain can rapidly condense around this nucleus.

**Fig. 3:**
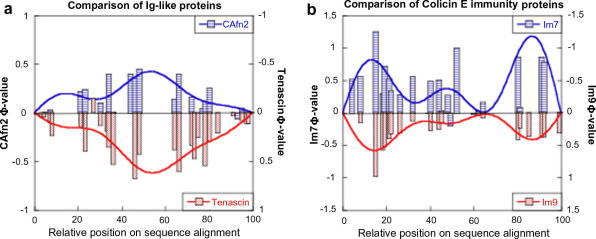
Comparison of homologous proteins. (a) Folding of two Ig-like domains, CAfn2 and TNfn3, both members of the fnIII superfamily. The pattern of Φ-values is extremely well conserved between the two domains implying equivalent folding pathways [18,19]. (b) Comparison of the folding of two colicin E immunity proteins. The pattern of Φ-values is extremely well conserved between the two proteins, despite the fact that Im9 folds with two-state kinetics whereas Im7 populates an on-pathway folding intermediate [29].

A sequence alignment from Pfam confirmed that the obligate folding nucleus is highly conserved within the Ig superfamily (e.g. I27), and is based around an invariant tryptophan residue located within the C strand. This result contrasts with that found for the fibronectin type III (fnIII) superfamily (e.g. TNfn3 and CAfn2), where the nucleus does not appear to have a fixed position within the hydrophobic core [19]. Nevertheless, the pattern of Φ-values is the same for TNfn3 and CAfn2, which leads to the conclusion that the two proteins have analogous transition state structures (Fig. 3).

There is also a high degree of similarity between the Φ-values in the Ig domain I27 and those in the fnIII domains TNfn3/CAfn2, especially when the effect of the different native state topologies is discounted. Taken together, these results provide strong evidence that the folding pathway is conserved between the two superfamilies, and that topology is the principal determinant of the folding of these complex Greek-key proteins. Experimental data on Ig-like domains have been used to develop algorithms to predict folding mechanisms within protein families [20].

Other proteins that appear to fold through conserved folding pathways include members of the SH3-domain superfamily [21–25], and some members of the ferredoxin-like fold [26,27].

### 2.2 A folding pathway is not defined by its kinetic intermediates

A detailed protein engineering study requires a significant input of time and expense, and it is much easier to attempt a comparison of folding pathways and mechanisms based solely on the kinetic behaviour of wild-type proteins. However, it is possible for two domains to exhibit different kinetic profiles and yet still fold through exactly the same pathway.

The best-known example of this type of behaviour is found for the homologous immunity proteins Im7 and Im9, where preliminary experiments showed that Im9 folds in a two-state manner, whereas Im7 populates an on-pathway intermediate [28,29]. Despite this difference, subsequent Φ-value analyses demonstrated that the two transition states were almost identical (Fig. 3), and provided strong evidence that the two proteins fold through the same pathway. This hypothesis was confirmed when Im9 was engineered to fold through an intermediate that displayed all of the structural characteristics of the Im7 intermediate [30].

An example that demonstrates that folding pathways can be conserved, even where folding intermediates differ, comes from the lipocalin superfamily. Wild-type IFABP folds through an intermediate with no observable secondary structure, whilst ILBP displays a folding intermediate with native-like secondary structure [31]. Interestingly, a single point mutation in IFABP causes this protein to fold through an intermediate that is identical to that of wild-type ILBP, whereas a single point mutation in ILBP leads to an intermediate that lacks any secondary structure [32]. Although there have been no further studies to confirm that the folding pathways of these two proteins are identical, the simple kinetic experiments are certainly not able to prove that they are different.

It is possible to understand how almost identical folding pathways can exhibit very different folding intermediates by considering that proteins fold by a diffusive process across a potential energy surface [33]. A folding pathway is thus described by the route that the polypeptide chain takes as it traverses the surface from the denatured to native states. The folding intermediates are represented by potential energy wells on this surface, and populated if these wells are sufficiently deep such that they form traps.

In the case of Im7 and Im9, which have been shown to follow the same route across the energy landscape, the intermediate well for Im9 is much shallower than that of Im7. As a result, Im7 populates a folding intermediate whereas Im9 does not, leading to a difference in kinetic behaviour [29]. By changing the amino-acid sequence of Im9, Radford and co-workers managed to increase the stability of the Im9 intermediate, without changing its position on the free-energy surface [30]. It has been suggested that if the traps exceed ∼3 kT then they will be sufficient to introduce detectable slowing of the folding [34–36].

For ILBP and IFABP, it is likely that one of these wells occurs early in the folding pathway, before the secondary structure has consolidated, and another occurs much closer to the native state. There is a single residue (at position 68 in IFABP, position 69 in ILBP), that controls the relative depths of these wells and can therefore switch the structure of the folding intermediate from denatured-like to native-like whilst retaining the same folding pathway.

Other proteins for which intermediates have been introduced into, or removed from, a folding pathway include Ig-like domains [37], cytochromes [38], and homeodomain-like bundles [39]. For a more thorough description of this area, see the review by Brockwell and Radford [40].

### 2.3 Proteins that fold with the same mechanism through different pathways

**Fig. 4:**
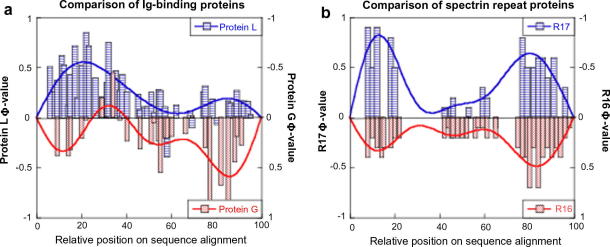
Comparison of the folding of homologous proteins with topologically symmetric native states. (a) Proteins L and G are both Ig-binding domains and exhibit a ‘breakdown of symmetry’ in their transition state ensembles. The pattern of Φ-values for Protein L is essentially a mirror-image of that found for Protein G [41,42]. (b) R16 and R17 are both spectrin repeat domains and fold through a transition state that is almost symmetrical. The pattern of Φ-values is well conserved between the two domains [45,81].

Proteins G and L are two homologous proteins from the topologically symmetrical Ig-binding superfamily. A direct comparison of their Φ-value patterns reveals no correlation whatsoever and confirms that the two proteins fold through different pathways [41,42] (Fig. 4a). However, a visual inspection of the regions of high Φ-values mapped onto the native state structures (Fig. 1), reveals that the two are qualitatively similar and appear to be mirror-images of each other. In both cases, one of the β-hairpins is fully formed and is packing against the central α-helix whereas the other β-hairpin is almost completely disordered.

Theoretical studies disagree as to whether topologically symmetrical proteins should fold through a transition state that is also symmetrical [43,44]. Whilst it could be argued that the transition state of the spectrin repeat domains R16 and R17 is symmetrical [45,46], (Fig. 4b), most other proteins in this class display asymmetric patterns of Φ-values [8,47–49].

Proteins that exhibit a ‘breakdown of symmetry’ within the transition state ensemble are particularly interesting, since they are able to highlight determinants of the folding mechanism beyond the native state topology [41]. For example, the first β-hairpin of Protein G is destabilized by several sterically unfavourable φ/ψ angles whereas the second β-turn contains a strongly stabilising side chain: main chain hydrogen-bond [42]. Baker and co-workers used computer-based redesign to remove the steric clashes from the first β-turn, and also removed the side chain: main chain hydrogen-bond from the second hairpin. The resulting protein, termed NuG2, folded 100 times faster than the wild-type and, more importantly, folded through a transition state structure analogous to that of Protein L [50].

It should be made clear that these results do not suggest that Proteins G and L fold through the same pathway. Rather, they demonstrate that symmetrical proteins have access to two or more distinct pathways to the native state. The results also highlight that, in some cases, the favored route can be determined by only minor differences in the free-energies of the various transition states.

These conclusions are further supported by studies on the repeat protein myotrophin, which comprises four ankyrin repeats and is highly symmetric [51]. A Φ-value analysis of this protein indicated that the folding nucleus is situated within the C-terminus of the protein (repeats 3 and 4), consistent with results from other ankyrin proteins [52]. However, several mutations within the folding nucleus produced unusual anti-Hammond behaviour, and suggested the presence of parallel folding pathways.

To test this hypothesis, Lowe and Itzhaki first destabilized the N-terminus of the protein by making the mutation A9G in the first ankyrin repeat. In the context of this mutant, the previously unusual behaviour seen in the C-terminus was abrogated [51]. These results indicated that myotrophin is able to fold from both the N- and C-termini, with the C-terminal nucleus being favored in the wild-type protein. When a sufficiently destabilizing mutation is made in the wild-type nucleus, the flux through the pathways is altered so that most proteins now fold using the N-terminal nucleus: this leads to the anti-Hammond behaviour. By ‘pre-destabilizing’ the N-terminal nucleus, this switch is prevented and C-terminal mutations provide the expected results.

It is worth stressing that not all symmetrical proteins have easy access to multiple folding pathways. For example, the B domain of Protein A has been the subject of more than a dozen folding simulations, none of which are able to reproduce the experimentally determined folding pathway in its entirety [53,54]. It was suggested that these discrepancies may be due to the symmetry of the protein, since multiple folding pathways would complicate any comparison between experimental and simulated results [55,56]. In particular, it was argued that an N-terminal nucleus would be more stable at lower temperatures (i.e. under experimental conditions), whereas a C-terminal nucleus would dominate at higher temperatures (where unfolding simulations are performed). Sato and Fersht characterised the folding transition state of Protein A as a function of temperature from 25 to 60 °C but the pattern of Φ-values was essentially unchanged, leading the authors to conclude that this protein folds through a single dominant folding pathway with an asymmetric transition state ensemble [54]. No moderate change in solvent conditions or amino-acid composition is able to force the protein to fold through the C-terminal nucleus.

### 2.4 Proteins fold through a continuum of mechanisms

Four members of the homeodomain-like superfamily have been studied by both experiment and simulation. Excluding proline phases, hTRF1, hRAP1 and c-Myb all exhibit two-state folding kinetics with no detectable intermediate [47]. Conversely, engrailed homeodomain (EnHD) displays multiphasic kinetics and folds through an on-pathway intermediate that comprises extensive secondary structure but few tertiary contacts [7].

To further investigate these differences, a limited protein engineering study was performed on EnHD and c-Myb. The Φ-values for EnHD, especially the alanine → glycine scanning results, are most consistent with a framework (diffusion–collision) mechanism of folding [47] (Fig. 2). In contrast, the Φ-values for c-Myb suggest that it folds through a transition state with an extended, loosely packed hydrophobic nucleus, which is more typical of a nucleation–condensation mechanism [47].

All-atom molecular dynamics simulations were able to reproduce the Φ-value patterns of EnHD and c-Myb, agreed with their experimentally determined unfolding rates, and showed the expected EnHD folding intermediate. Interestingly, these simulations also predicted the existence of an intermediate on the c-Myb folding pathway, which contrasted with the experimental results. However, this intermediate was later shown to be a real feature of the folding pathway when Fersht and co-workers engineered a point mutant that folded with three-state kinetics [39]. This excellent agreement between *in vitro* and *in silico* work was used to validate the MD methods, and allowed the authors to draw conclusions from simulations of hTRF1 without experimental benchmarking. A detailed analysis of the unfolding trajectories of EnHD, c-Myb and hTRF1 showed that all three proteins fold through transition state ensembles that are based around a conserved folding nucleus; however, the proteins differ in the order in which native interactions are formed. The transition state for EnHD occurs when pre-formed helices dock together in a final rate determining step. In this case, secondary structure formation precedes tertiary structure formation. At the other extreme, the transition state of hTRF1 appears to be an expanded version of the native state with very few interactions that are fully formed: secondary and tertiary interactions form concomitantly. Finally, the transition state of c-Myb exhibits a degree of structure formation that is intermediate between the two other proteins.

This discovery that nucleation–condensation and diffusion–collision are actually different manifestations of a ‘common unifying folding mechanism’ is further supported by studies on the PDZ2 domain. This protein folds through two sequential transition states, both of which have been characterised by Φ-value analysis [57]. The first transition state exhibits a pattern of Φ-values that is consistent with a nucleation–condensation mechanism: moderate values in the folding nucleus, but uniformly low values elsewhere. The second transition state, however, shows discrete blocks of high Φ-values, which correspond to the native secondary structural elements. This pattern is far more consistent with a framework mechanism.

The authors use these results to propose that small globular proteins fold through three discrete phases: (a) formation of a weak nucleus that sets up the overall native state topology, (b) global compaction of the entire polypeptide chain in which secondary structural elements condense, and (c) consolidation of the remaining tertiary interactions. For most proteins, the rate determining step occurs in the second phase of the folding process and thus the Φ-values indicate a nucleation–condensation mechanism. Where there are unusual circumstances, for example high innate helical propensity, it is actually the third phase that becomes rate determining, and the protein engineering studies reveal extreme Φ-values consistent with a framework mechanism.

A ‘continuum of mechanisms’ has also been seen in the peripheral subunit-binding domain (PSBD) superfamily, where there is a change from two-state to multiphasic kinetics as the denatured state becomes more structured [58]. Protein engineering studies of the mesophilic homologue BBL [59], the thermophilic E3BD [58], and the hyperthermophilic POB [60], reveal that all three proteins fold through a nucleation–condensation mechanism, with few extreme Φ-values. However, there is a ‘slide’ from BBL, which exhibits a very diffuse transition state, through E3BD to POB, which displays a highly polarised transition state structure. As with the homeodomain superfamily, this phenomenon is linked to inherent secondary structural propensity [59].

### 2.5 Protein folding is a balance between entropy and enthalpy

A protein engineering study is used to probe the contribution of individual amino-acid side-chains to the structure of the transition state ensemble. The desired outcome of a point mutation is a transition state that is unchanged in structure, but simply has a lower enthalpy of contacts and hence a higher free-energy. Less often, an alternate folding pathway becomes more energetically favourable and there is a marked change in the structure of the transition state ensemble (e.g. myotrophin [51]). Both of these scenarios are informative, and can be used to identify the enthalpic reasons for a particular folding pathway. However, enthalpy is only half the story, and for a complete understanding of protein folding it is necessary to assess the entropic reasons for the structure of the transition state. For this reason, circular permutation is an excellent tool for the study of protein folding pathways, since it changes the entropic cost of a particular residue contact without affecting its enthalpic contribution.

**Fig. 5:**
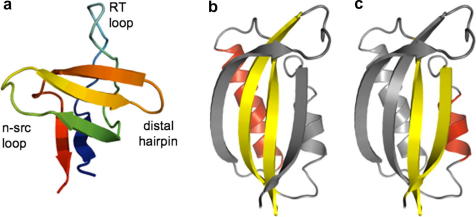
Examples of proteins studied by circular permutation. (a) α-Spectrin SH3-domain: cutting the RT loop does not result in a change in transition state structure (centred around the 3-stranded central sheet and the distal hairpin and n-src loop). Cleaving the distal hairpin, however, results in a different, more diffuse nucleus [61,62]. (b and c) Two different nucleation motifs within the ferredoxin-like fold. (b) Wild-type S6_T_ and U1A predominantly fold using a nucleus comprising the two central β-strands and the longer α1 helix [13,64]. (c) S6_A_, ADA2h and circular permutants of S6_T_ fold using an alternative two-strand-helix nucleation motif [27,64,65].

Early studies on the folding of α-spectrin SH3 revealed a folding nucleus that was based around interactions between two β-hairpins known as the ‘RT loop’ and the ‘distal loop’ (Fig. 5a) [61]. To investigate whether the transition state was dominated by entropic factors, Serrano and co-workers created two circular permutants by fusing the wild-type N- and C-termini, and cleaving the backbone in either the RT loop or the distal loop. When the RT loop was cleaved, the pattern of Φ-values was essentially unchanged from wild-type, and the RT contacts were still packed together in the transition state ensemble. However, when the distal loop was cleaved the folding nucleus moved so that it comprised a third hairpin, known as the ‘n-Src loop’, and the new hairpin formed between the old N- and C-termini. Neither the distal contacts, nor the RT contacts, were involved in this new transition state ensemble.

These results suggest that the SH3 topology contains two potential nucleation motifs: RT loop plus distal loop, and n-Src loop plus terminal loop. To explore which nucleus would dominate if all four hairpins were available simultaneously, a further study was conducted which circularised the SH3-domain using a disulphide crosslink [62]. In this case, the protein folded through a very diffuse transition state ensemble, suggesting that folding is able to proceed from either nucleus. These studies provide a nice illustration of differentiating between contacts that are recruited for their high enthalpic contribution (i.e. those in the RT loop), and contacts that are engaged primarily because of the low entropic cost of their formation (i.e. those in the distal loop and the terminal loop).

A detailed entropy analysis was also performed on the ferrodoxin-like ribosomal protein S6_T_. As with SH3, a circular permutant was created by fusing the N- and C-termini and cleaving the backbone in the region of the original folding nucleus. In this case, the globally diffuse transition state of the wild-type became an extremely polarised transition state in the circular permutant [63]. The authors explained this change by noting that there is a biased distribution in the contact energies of the wild-type protein: interactions between residues that are far apart in sequence, and therefore incur a high entropic penalty, are much stronger than local contacts, which incur a low entropic penalty. This entropy:enthalpy compensation results in a transition state that is globally diffuse. In the circular permutant, however, this balance is disrupted, leading to a very polarised transition state ensemble.

The entropy analysis of S6_T_ was later extended to incorporate two further circular permutants. In both cases, the folding nucleus moved within the structure in a manner that could be predicted by loop entropy calculations [64]. Interestingly, the transition state of each of the four permutants comprised two β-strands and an α-helix, although the exact appearance and location of the nucleus varied with the chain connectivity. Oliveberg and co-workers suggested that ferrodoxin-like proteins have access to two potential folding pathways, a hypothesis supported by their analysis of the Φ-value patterns of three homologues of S6_T_: two from the same laboratory (U1A [13], S6_A_ [65]) and another from the Serrano lab (AdA2h [27]). They found that whilst wild-type S6_T_ and U1A predominantly fold through the “ββα1” nucleation motif, ADA2h, S6_A_ and the three circular permutants of S6_T_ fold through the alternative “ββα2” nucleation motif (Fig. 5b and c).

This ‘two-strand-helix’ motif appears to be a common nucleation mechanism, and has be identified as the folding nucleus of several proteins, including: CI2, ubiquitin, U1A, L23, CheY and ctAcP [66]. A ‘two-helix-strand’ nucleation motif has also been identified, and is used to nucleate folding in the LysM domain [49] and the FF domain [67]. Oliveberg and co-workers describe these nucleation motifs as ‘foldons’, and suggest that they correspond to the smallest elements with sufficient stabilising interactions to overcome their loss in chain entropy. The authors state that it is no coincidence that these foldons correspond to the size of the smallest globular proteins [66]. Studies of circular permutants of PDZ domains suggest that early events are particularly sensitive to chain connectivity, so that different competing nuclei are selected in different permutants, whereas the late transition states of the permutants are robust [68].

The detailed analyses of the ferrodoxin-like proteins were extended to other homologues in order to identify the number and arrangement of foldons within other protein topologies [66]. The authors noted that where a protein consists of a single foldon, the pathway is robust and there is little evidence of Hammond or anti-Hammond behaviour. Such proteins include CI2 and LysM, both of which have a transition state that is unaffected by even large scale changes such as circularisation [49,69]. These proteins can be described as ‘ideal’ two-state folders. In contrast, where a protein comprises spatially separate foldons, the global cooperativity of the folding pathway is broken and kinetic intermediates may accumulate. Examples include the lipocalins [32], the cell-cycle regulatory proteins [70] and human serum albumin [71]. Finally, but most interestingly, where a protein contains multiple overlapping foldons then evolution can ‘tune’ the nucleation potentials of the submotifs so that the structural ordering of one motif is able to automatically initiate the folding of others. This is the case for the ferrodoxin-like proteins, where the two foldons share a common β-strand (Fig. 5). In these cases, the order of folding events is not critical and mutations are able to induce transition state shifts both along (Hammond behaviour) and across (anti-Hammond behaviour) the energy landscape [13,64,66].

It is worth noting that the nuclei of the topologically complex Greek-key proteins comprise four elements of secondary structure (Fig. 1a), rather than the three elements of the two-helix-strand or two-strand-helix motifs. Since some members of the Ig-like fold display significant pathway malleability [72], it would be interesting to elucidate whether their folding nuclei contain two overlapping three-strand foldons, or whether the more intricate topology requires a larger nucleation motif. Does foldon size increase with complexity? This idea is supported by the observation that in simple 3-helix bundles, such as the engrailed homeodomains, Protein A and spectrin domains, only two of the helices are important for nucleation.

### 2.6 The importance of gross topology versus local interactions

Although it is clear that local interactions are key to determining the precise folding pathway and mechanism, it is the gross topology that initially creates the potential repertoire of accessible folding routes. Take, for example, Proteins L and G: the topology of the ubiquitin-like fold determines the necessary structure of the transition state, but its symmetry provides two alternatives. It is the finer detail of the primary amino-acid sequence that determines which of the two nucleation motifs is the more stable. Hence Proteins L and G fold through entirely different transition states. Another example is the topology of the ferrodoxin-like fold, which provides two overlapping ββα nucleation motifs. Although individual members of this fold exhibit very different transition state structures, they are all explained by simply considering the relative free-energies of the two competing nuclei.

Since the native state determines the potential nucleation sites, it is no surprise that folding rates can be predicted from gross topology. In particular, long-range order [73] is extremely good at predicting the folding rate of a protein based solely on its topological complexity. The findings indicate that α-helical proteins, with low complexity, tend to fold much faster than β-sheet proteins or mixed αβ proteins, which exhibit high topological complexity. As might be expected, there is significant scatter to the correlation and the authors mention that topology merely sets upper and lower bounds to the folding rate. It is the unique distribution of contacts within the native state of a protein that ultimately decides the exact folding rate.

The death domain from FADD provided a unique opportunity to investigate the folding of an all-α protein that nevertheless possessed a high topological complexity. This domain essentially comprises two orthogonally packed three-helix bundles, although the topological arrangement of the six helices results in a complex Greek-key topology [74], as seen for the Ig-like domains. The authors considered two possibilities: either the secondary structure dominates, in which case the two three-helical bundles should fold first and then pack together; or the topology dominates, in which case the domain should mimic the Ig-like domains and the four central helices should pack first using key-residues.

A detailed Φ-value analysis revealed that the two edge helices do not participate in the transition state, and are essentially unstructured. This mimics the Ig-like domains where the edge β-strands show Φ-values of zero. In contrast, the four central helices show considerable native-like structure, with a nucleus based around several long-range packing interactions. It is clear that the death domain folds through a transition state that resembles the all-β Ig-like proteins and that, in this case, the complex topology of the Greek-key motif supersedes the secondary structural makeup of the death domain.

## 3 Insights from theoretical analyses of the folding of homologous proteins

Energy landscape theory has been a powerful tool for informing our understanding of protein folding mechanisms. It is a fundamental tenet of such studies that evolution has resulted in proteins which have funnelled energy landscape, which guide the proteins towards the native state [75]. These energy landscapes are generally accepted to be smooth, or non-frustrated, largely lacking kinetic traps which would slow folding, and avoiding alternative, stable non-native forms. Interestingly, however, it does not seem that the residues that constitute the folding nucleus of proteins with conserved folding mechanisms, are themselves specifically conserved (although some investigators suggest this is the case, this is not a general rule) [76,77]. Rather, proteins have evolved such that native interactions are far more favourable than non-native ones.

Recently Cho et al. [78] have revisited energy landscapes in the light of the continuing body of knowledge which has come from studies of the folding of related proteins. In particular they address the questions thrown up by such studies: how can failure of topology-based models to predict folding mechanisms be reconciled with funnelled landscape theory? The answer, of course, lies in the balance between the enthalpy gain in forming specific contacts with the entropy cost of forming those contacts. In particular their analysis suggests that for all-α proteins the principle determinant of folding pathway is the enthalpic gain from specific side chain interactions, because the entropic costs of folding are rather uniform across the structure. Thus, the analysis suggests that all-α proteins are more likely to have folding pathways which vary from one family member to another. For all-β proteins, or for α proteins with more complex topologies, folding pathways are more likely to be conserved – that is, less likely to be disrupted by sequence changes, which is precisely what is observed in the experimental studies.

In light of the success of landscape theory, it is perhaps not surprising that simple metrics, such as contact order, or long-range order can relate the protein topology (in terms of local vs. non-local contacts) to the rate at which structure is formed. Essentially this demonstrates that chain entropy plays a key role in determining the energetics of folding. Such straightforward relationships have lead to the success of relatively simple folding models, such as that proposed by Munoz and Eaton to predict folding kinetics [79]. Further, simplified topology-based models can often recapitulate many of the features of protein folding – simple Go-like models of proteins are often sufficient to predict folding mechanisms. Thus, since knowledge of the native topology is sufficient for predicting the folding properties of proteins this suggests that transition state properties reflect the native state.

## 4 Insights from chimeric proteins

There may be a simple explanation why some proteins have folding rate constants which cannot be well predicted by simple models. Ig-like domains, for example, which all have approximately the same relative contact order, fold with rate constants which span >5 orders of magnitude. Interestingly, however, their folding rate constants correlate strongly with stability. From this one can simply conclude that the transition state stability is determined essentially by the same interactions that impart stability on the native state, suggesting that the height of the energy barrier is dominant. Interestingly in the same family, the more stable members of the family have stable folding intermediates – presumably a similar reasoning applies. Since these proteins all fold by the same nucleation–condensation mechanism with a nucleus forming in the same region of the protein in all cases, the most stable proteins will have folding intermediates which are stabilised sufficiently to be populated. As was the case for the immunity proteins, lack of a folding intermediate in some members of the family does not infer a different pathway, simply different stabilities of species on the folding pathway.

It is where these simple models fail to predict the differences in folding behaviour of homologous proteins that the questions get more interesting. Our laboratory has been studying the folding of the apparently simple 3-helix bundle spectrin fold. The aim of this study was to compare the folding of a simple helical fold with that of the complex all-β Greek-key Ig-like domains. Three domains were investigated in detail, R15, R16 and R17 which have a ∼30% pair-wise sequence identity. What was remarkable about these three proteins was the three orders of magnitude difference in folding and unfolding rate constants – yet the proteins had the same stability [80]. Here, serendipity plays a part. Had the difference in folding rate constants been a “mere” 100-fold or so, this might not have borne investigation. Examination of a contact order plot shows that a 2-order of magnitude scatter is nothing remarkable. Yet these same plots show that the slow folding proteins fall way below the expected value. Simple explanations (a significantly more structured transition state in the fast folding proteins) did not explain the data, and simulations threw no light [45,46,81]. An intriguing possibility was that the slow folding proteins had a rugged, or frustrated energy landscape and that kinetic traps were responsible for the slow folding behaviour. Experimental evidence for “internal friction” playing a role in protein folding has been hard to find, except in the case of very fast folding proteins and for reorganisations in essentially folded states. Following the approach of Eaton and co-workers, we were able to show experimental evidence to suggest that the slow folding is due, at least in part, to frustration in the energy landscape of the slow folding domains [82].

What was important in the next step was the opportunity that the study of related proteins allows. First the core of the fast folding protein was grafted into the slow folding domains – the essentially identical structure allowed stable proteins to be produced. These folded rapidly with a concomitant decrease in landscape roughness. This has never been observed previously for a protein folding on a relatively slow ms-s timescale. The folding mechanism also changes from a strictly framework mechanism (where secondary structure formation precedes tertiary packing) towards a nucleation–condensation mechanism (as found in the fast folding domains). Interestingly, the studies of the families of homeodomains (also 3-helix bundles) gave quite a different picture – the fast folding family members fold via a framework mechanism – packing of pre-formed helices lead to fast folding. This comparison between two such studies of the folding of homologous proteins was essential in allowing us to propose that the search for the correct register to pack the long helices of the spectrin domains results in transient misdocking of the helices and slower folding – in contrast, the short helices of the homeodomains face no such challenge.

Such a mix-and-match approach is not new: for instance, in a recent study of the role of folding intermediates in folding of Ig-like domains Buchner and co-workers used a similar grafting methodology, transplanting elements of structure from an antibody domain into the homologous β(2) microglobulin [83]. This chimeric protein had significantly reduced amyloidogenicity.

## 5 The future of the “fold approach”

From our analysis of what has been achieved is possible to argue that it is no longer enough to just investigate any other family, the choice of a family of proteins to be studied should be designed to address specific questions. The caveat, of course, is that unusual results may be found in an apparently simple system (as was the case for the spectrin domains).

### 5.1 Selecting proteins for study

The use of theory and databases can facilitate the choice of family members to be investigated. Again, in the homeodomain study, the first examples, EnHD and c-Myb, show very different helical propensity in AGADIR and the “extreme” protein, hTRF1, was chosen on the basis of its AGADIR profile. Similarly, sequence databases such as Pfam can allow highly similar or highly diverse members of the same family to be selected, and SCOP allows proteins with the same fold but in different, possibly unrelated superfamilies to be investigated. An exciting development is the dynameomics projects from the Daggett lab, which aims to investigate the (un)folding of a representative member of all structural families [84].

A further opportunity, as theoretical investigations of related proteins advance, is the possibility to use theory to inform experiment. Recent studies of the immunity proteins have lead Wolynes and co-workers to propose mutations to engineer the intermediate in Im7 [36], for example.

Of course, clear stated aims of such studies must be paramount. As we have said, there are still outstanding questions about the role of symmetry, topology and of sequence conservation – careful selection of families from the databases should allow us to address these very explicitly.

### 5.2 Comparisons within a fold/comparisons between folds

The questions to be considered are: how many proteins do we need to study? How dissimilar (in sequence) should they be? Are highly conserved families less useful? What structural features should the protein(s) of interest have?

The examples we have analysed would suggest that the more members of a family which are compared the more likely it is that interesting, unexpected and challenging behaviour would be observed. Had only a single member of the engrailed homeodomain family been studied then the slide between mechanisms would not have been observed. For some questions, proteins with very diverse sequences might be appropriate, but a recent observation that two proteins with ∼88% sequence identity have different structures suggests that differences between highly related proteins could be interesting [3]. The clear advantage of choosing proteins with the same fold but in different superfamilies allows folding and function to be separated.

Another example of such a deliberate choice was the selection of the study of the folding of a death domain [74]. In this case a comparison of the folding of topologically complex all-β Greek-key Ig-like domains, with topologically simple, 3-helix bundle spectrin domains raised the question of whether the real differences in folding behaviour were a function of topological complexity or secondary structure content. The studies of the all-α Greek-key protein, selected from inspection of the SCOP database, allowed us to suggest that topology is the principal determinant of folding mechanisms.

A challenging field is the analysis of the folding of membrane proteins, which fall outside the scope of this article – yet even here the study of structurally similar proteins has been informative. Membrane proteins essentially fall into two classes – β-barrels and helix bundles. Comparative studies of the folding within classes and between classes are underway (see, for example, [85,86]). Current data suggest that there will be generic folding mechanisms for the different classes: insertion and packing of pre-formed helical elements or concerted folding and insertion of β-barrels [87,88]. Particularly exciting is the introduction of Φ-value analysis into the studies of membrane protein folding [89,90].

### 5.3 Common folds as the basis for design

Studies of the folding of homologous proteins can identify residues important for folding and thus identify non-essential regions of the structure amenable to design. Nature is there before us: in the Ig-like domains, for instance, residues important for folding are buried residues at the centre of the core, in the four central strands. This has allowed other regions of the protein (surface of the sheets, peripheral strands and loops) to evolve a remarkable array of functions (such as binding or mechanical strength). An example of rational design is the engineering of stable repeat proteins of varying size which have novel binding specificities [91,92]. Again, the choice of protein system was clever and important for the success of these studies. Design of stable consensus repeat proteins allowed free engineering of binding surfaces.

In conclusion, we suggest that whilst there are still interesting questions to be addressed in the protein-folding field, then comparative studies of structurally related proteins will play an important role.

## Figures and Tables

**Table 1:** Protein folds where the folding of homologous proteins has been studied.

Class (fold)	Superfamily	Protein (species)	Method of investigation	PDB code	Experimental references	Comparative references^a^
All-α (Acyl-CoA binding protein-like)	Acyl-CoA binding protein	ACBP (Cow)	Φ-Value analysis	2ABD	[93,94]	[94]
		ACBP (Rat)	WT kinetics	2ABD^b^	[93]	
		ACBP (Yeast)	Φ-Value analysis	2ABD^b^	[94]	

All-α (Acyl carrier protein-like)	Colicin E immunity proteins	Im7 (*E. coli*)	Φ-Value analysis	1AYI	[28]	[29]
		Im9 (*E. coli*)	Φ-Value analysis	1IMQ	[29,30,95]	

All-α (Cytochrome *c*)	Cytochrome *c*	Cytochrome *c* (Horse)	Hydrogen exchange	1HRC	[96]	[97]
		Cytochrome *c*2 (*R. capsulatus*)	WT kinetics	1C2R	[98]	
		Cytochrome *c*551 (*P. aeruginosa*)	Minimal Φ-value analysis	2PAC	[99]	
		Cytochrome *c*552 (*H. thermophilus*)	WT kinetics	1AYG	[100]	
		Cytochrome *c*552 (*T. thermophilus*)	WT kinetics	1C52	[101]	
		Mitochondrial cytochrome *c* (Yeast)	WT kinetics	1YCC	[102]	

All-α (Four-helical up-and-down bundle)	Cytochromes	Cytochrome *b*562 (*E. coli*)	Hydrogen exchange	1APC	[103]	
	FKBP12-rapamycin-binding domain of FKBP-rapamycin-associated protein (FRAP)	FRB (Human)	WT kinetics	1AUE	[104]	

All-α (DNA/RNA-binding 3-helical bundle)	Homeodomain-like	DNA-binding domain of human telomeric protein hTRF1 (Human)	WT kinetics	1BA5	[47]	[47]
		En-Hd (Drosophila)	Φ-Value analysis	1ENH	[7,105]	
		c-Myb DNA-binding domain (Mouse)	Φ-Value analysis	1IDY	[47]	
		Rap1 (Human)	WT kinetics	1FEX	[47]	

All-α (Globin-like)	Globin-like	Leghemoglobin (Soybean)	Hydrogen exchange	1FSL	[106]	[106]
		Myoglobin (Sperm whale)	Hydrogen exchange	1A6M	[107]	

All-α (peripheral subunit-binding domain of 2-oxo acid dehydrogenase complex)	Peripheral subunit-binding domain of 2-oxo acid dehydrogenase complex	E3 binding domain of dihydrolipoamide acetyltransferase [E3BD] (*B. stearothermophilus*)	Φ-Value analysis	1EBD	[58]	[59]
		E3-binding domain of dihydrolipoamide succinyltransferase [BBL] (*E. coli*)	Φ-Value analysis	1BBL	[59]	
		POB (*P. aerophilium*)	Φ-Value analysis	1BBL^b^	[60]	

All-α (ROP-like)	ROP protein	ROP (*E. agglomerans*)	WT kinetics	1ROP^b^	[4]	[4]
		ROP (*E. coli*)	WT kinetics	1ROP	[4]	
		ROP (*P. vulgaris*)	WT kinetics	1ROP^b^	[4]	

All-α (spectrin repeat-like)	Spectrin repeat	Alpha chain R15 (Chicken)	Φ-Value analysis	1U5P	[46]	[46]
		Alpha chain R16 (Chicken)	Φ-Value analysis	1CUN	[81]	
		Alpha chain R17 (Chicken)	Φ-Value analysis	1CUN	[45]	

α/β (α/β knot)	α/β knot	YbeA (*E. coli*)	Φ-Value analysis	1NS5	[108]	[108]
		YibK (*H. influenzae*)	Φ-Value analysis	1J85	[109]	

α/β (Dihydrofolate reductase-like)	Dihydrofolate reductase-like	Dihydrofolate reductase (*E. coli*)	WT kinetics, Ligand binding	1RA9	[110]	[110]
		Dihyrofolate reductase (*L. casei*)	WT kinetics, Ligand binding	3DFR	[110]	
		Dihydrofolate reductase (Human)	WT kinetics, Ligand binding	1KMV	[110]	

α/β (flavodoxin-like)	CheY-like	CheY (*E. coli*)	Φ-Value analysis	1EAY	[111]	[112]
	Flavoproteins	Apoflavodoxin (*A. vinelandii*)	WT kinetics	1YOB	[113]	
		Flavodoxin (*Anabaena pcc 7119*)	Φ-Value analysis	1FTG	[112]	

α/β (Phosphoglycerate kinase)	Phosphoglycerate kinase	Phosphoglycerate kinase (*B. stearothermophilus*)	Minimal Φ-value analysis	1PHP	[114]	
		Phosphoglycerate kinase (Yeast)	WT kinetics	3PGK	[115]	

α/β (RNase-H-like Motif)	RNase-H-like	RNase-H (*E.coli*)	Hydrogen exchange Limited mutagenesis	1F21	[168,169]	
		RNase-H (*T. thermophilus*)	Hydrogen exchange	1RIL	[170]	[170,171]
		RNase-H (*C. tepidum*)	WT kinetics	3H08	[171]	

α/β (TIM β/α-barrel)	Ribulose-phosphate binding barrel	Trptophan synthase α-subunit [αTS] (*E. coli*)	WT kinetics	1V7Y	[116]	[117]
		Indole-3-glycerophosphate synthase [sIGPS] (*S. solfataricus*)	WT kinetics, Hydrogen exchange	1IGS	[118,119]	

	Xylose isomerase-like	IOLI (*B. subtilis*)	WT kinetics	1I60	[117]	

α + β (Ferredoxin-like)	Acyl-phosphatase-like	AcP (Human)	Φ-Value analysis	1APS^b^	[26,120]	[26,65,121]
		HypF (*E. coli*)	WT kinetics	1GXU	[121]	
	Protease propeptides/inhibitors	Procarboxy-peptidase A2 (Human)	Φ-Value analysis	1O6X	[27]	
	Ribosomal protein S6	S6 (*A. aeolicus*)	Φ-Value analysis	2J5A	[65]	
		S6 (*T. thermophilus*)	Φ-Value analysis	1RIS	[69]	
	RNA binding domain (RBD)	U1A (Human)	Φ-Value analysis	1FHT	[13]	

α + β (β-hairpin-α-hairpin repeat)	Ankyrin repeat	AnkyrinR D34 (Human)	Minimal Φ-value analysis	1N11	[122]	[123]
		Ankyrin repeats in tumor suppressor p16 (Human)	Φ-Value analysis	1BI7	[52]	
		Cell-cycle inhibitor p19ink4D (Human)	WT kinetics	1BD8	[124]	
		Myotrophin (Rat)	Φ-Value analysis	2MYO	[51]	
		Neurogenic locus notch receptor domain (Drosophila)	Minimal Φ-value analysis, WT redesign	1OT8	[125,126]	

α + β (Cell-cycle regulatory proteins)	Cell-cycle regulatory proteins	CksHs1 (Human)	Φ-Value analysis	1BUH	[70]	[70]
		CksHs2 (Human)	WT kinetics	1CKS	[127]	
		Suc1 (*S. pombe*)	Φ-Value analysis	1PUC	[128]	

α + β (Lysozyme-like)	Lysozyme-like	Lysozyme (Hen Egg White)	WT kinetics	1E8L	[129]	[130]
		α-Lactalbumin (Bovine)	WT kinetics	1F6S	[130]	
		α-Lactalbumin (Goat)	Minimal Φ-value analysis, Hydrogen exchange	1HFY	[131,132]	[131]
		Milk lysozyme (Dog)	Hydrogen exchange	1EL1	[131,133]	

α + β (β-grasp: ubiquitin-like)	Immunoglobulin-binding domains	Protein G (*Streptococcus*)	Φ-Value analysis	2IGD	[42]	[42,134,135]
		Immunoglobulin light chain-binding domain of Protein L (*P. magnus*)	Φ-Value analysis	2PTL	[41]	
	Ubiquitin-like	c-Raf1 RBD (Human)	Φ-Value analysis	1RFA	[134,135]	
		Ubiquitin (Human)	Minimal Φ-value analysis	1UBQ	[136]	
		Ubiquitin (Yeast)	Φ-Value analysis	1Q0W	[137]	

All-β (Ig-like β-sandwich)	Fibronectin type III	CAfn2 (*B. circulans*)	Φ-Value analysis	1K85	[19]	[15,19]
		FnIII-9 (Human)	WT kinetics	1FNF	[138]	
		FnIII-10 (Human)	Φ-Value analysis	1FNF	[16]	
		TNfn3 (Human)	Φ-Value analysis	1TEN	[18]	
	Immunoglobulin	TI I27 (Human)	Φ-Value analysis	1TIT	[17]	
		CD2 (Rat)	Minimal Φ-value analysis	1HNG	[37]	
		Various antibody domains (V_L_, C_L_, C_H_2, C_H_3)	WT kinetics		[139–142]	[142]

All-β (Lipocalins)	Lipocalins	CRABP I (Mouse)	WT kinetics	2CBR	[143,144]	[32,143]
		CRBP II (Rat)	WT kinetics	1OPA	[143]	
		IFABP (Rat)	Minimal Φ-value analysis	1IFC	[31,32,143,145]	
		ILBP (Rat)	Minimal Φ-value analysis	1O1V^b^	[31,32]	

All-β (OB-fold)	Nucleic acid-binding proteins	Bc-Csp (*B. caldolyticus*)	Φ-Value analysis	1C9O	[146,147]	[146,148]
		CspA (*E. coli*)	Hydrogen exchange	1MJC	[149]	
		Bs-CspB (*B. subtilis*)	Φ-Value analysis	1CSP	[150]	
		Tm-Csp (*T. maritima*)	WT kinetics	1G6P	[148]	

All-β (PDZ domain-like)	PDZ domain-like	PDZ2 domain from PTP-BL (Mouse)	Φ-Value analysis	1GM1	[57]	[151]
		Third PDZ domain from synaptic protein PSD-95 (Rat)	Hydrogen exchange Φ-value analysis	1BE9	[151,152]	

All-β (SH3-like barrel)	Chromo domain-like	DNA-binding protein Sso7d (*S. solfataricus*)	Φ-Value analysis	1SSO	[153]	[21,153]
	SH3-domain	α-Spectrin SH3-domain (Chicken)	Φ-Value analysis, WT redesign	1SHG	[22,154]	
		Fyn proto-oncogene tyrosine kinase SH3-domain (Chicken)	Φ-Value analysis, NMR dispersion	1FYN^b^	[23,24,155,156]	
		Actin binding protein ABP1 (Yeast)	NMR dispersion	1JO8	[21]	
		Phosphatidylinositol 3-kinase SH3-domain (Cow)	WT kinetics	2PNI	[157]	
		c-src protein tyrosine kinase (Chicken)	Φ-Value analysis	1SRM	[25]	

All-β (WW domain-like)	WW domain	Formin Binding Protein 28 (Mouse)	Φ-Value analysis	1E0L	[8,158]	
		Mitotic rotamase PIN1 (Human)	Φ-Value analysis	1PIN	[48,159]	
		Yap65 WW domain (Human)	WT kinetics	1JMQ	[160]	

Coiled coil proteins (Parallel coiled-coil)	Leucine zipper domain	GCN4 (Yeast)	WT kinetics, Minimal Φ-value analysis	2BNI	[161,162]	
		c-Jun (Human)	Dimer thermodynamics	1JUN	[163]	
		c-Fos (Human)	Dimer thermodynamics	1FOS	[163]	

a Comparative references are those in which the folding mechanisms/pathways of homologous proteins are compared and discussed.

b In these cases, there is no structural information available for the protein that was studied. The PDB code of a close homologue is given instead.
